# Tissue adaptation of eosinophils

**DOI:** 10.1093/jleuko/qiag064

**Published:** 2026-05-18

**Authors:** Clotilde Marie Lacroix, Kalyani Kulkarni, Nicola Laura Diny

**Affiliations:** Institute of Clinical Chemistry and Clinical Pharmacology, University Hospital Bonn, University of Bonn, Venusberg-Campus 1, Bonn 53127, Germany; Institute of Clinical Chemistry and Clinical Pharmacology, University Hospital Bonn, University of Bonn, Venusberg-Campus 1, Bonn 53127, Germany; Institute of Clinical Chemistry and Clinical Pharmacology, University Hospital Bonn, University of Bonn, Venusberg-Campus 1, Bonn 53127, Germany

**Keywords:** eosinophils, metabolism, microenvironment, tissue adaptation, transcriptional regulation

## Abstract

Eosinophils are increasingly recognized as tissue-resident immune cells that take on diverse functions. Recent studies have shed light on the pathways that result in tissue-specific eosinophil phenotypes. In this review, we discuss the process of tissue adaptation covering the local environmental cues that trigger adaptation, the transcriptional reprograming that occurs in response, and the phenotypic, metabolic, and functional adaptation that leads to varied eosinophil populations across the body. We further examine the relative impact of trained immunity and systemic factors versus local signals on eosinophil plasticity in the context of homeostasis and inflammation.

## Teaser

Tissue adaptation of eosinophils is triggered by local environmental factors and involves a network of regulators that govern transcriptional reprograming. This leads to the acquisition of new tissue-specific phenotypes and functions.

## Key Concepts

- Eosinophils undergo tissue adaptation in response to local environmental triggers, such as cytokines, growth factors, dietary or microbial factors, or cell–cell/cell–matrix interaction.
- Tissue adaptation is controlled by a network of regulators and leads to transcriptional and, likely, epigenetic reprograming.
- Tissue adaptation occurs gradually over time, during which eosinophils may pass through multiple niches within the tissue and may transition through several phenotypic states.
- Tissue-adapted eosinophils take on distinct phenotypes and tissue-specific functions.
- Inflammation and infection can alter local and systemic signals, leading to a change in eosinophil functions.

## Open questions

- Which factors in the microenvironment trigger the adaptation of eosinophils in different tissues?
- What are the regulatory networks of transcription factors governing eosinophils adaptation in each tissue?
- What is the relative importance of systemic versus local factors for eosinophil function?
- Is the tissue-adapted eosinophil phenotype stable or can it be reversed or altered in the context of infection or inflammation?
- Can the tissue adaptation pathways identified in animal studies be translated to humans and be exploited therapeutically in eosinophil-associated diseases?

## 1. Introduction

Tissue adaptation describes how immune cells acquire organ- and niche-specific phenotypes and functions, allowing them to meet the distinct structural, metabolic, and microbial challenges of each tissue. This process, studied mostly in T cells, macrophages, and innate lymphoid cells, describes how local cues reshape immune cell identity, positioning them as integral components of tissue physiology.^1–3^ A variety of signals such as cytokines, growth factors, metabolites, microbiota-derived products, and stromal and neural inputs converge to induce unique transcriptional, epigenetic, and metabolic programs in tissue-resident immune populations. Thereby, immune cell function is tailored to the needs of each organ.

Eosinophils have emerged as highly adaptable granulocytes that not only participate in type 2 immune responses in allergic asthma and host defence against parasitic infections but also execute diverse homeostatic functions specific to each tissue, as has been excellently reviewed recently.^4,5^ During development in the bone marrow, eosinophil maturation is influenced by type 2 cytokines, in particular IL-5 along with IL-3, IL-33, and granulocyte-macrophage colony-stimulating factor (GM-CSF). These promote the proliferation of eosinophil progenitors and eosinophil survival. During this developmental phase, eosinophils also synthesize and store large amounts of pre-formed granule proteins like major basic protein, eosinophil peroxidase, or eosinophil-derived neurotoxin, as well as other mediators.^6,7^ Mature eosinophils are released from the bone marrow, from where they migrate into tissues and to sites of infection or allergic inflammation. Upon activation, eosinophils undergo degranulation, releasing granule contents into the local environment. In addition to the granule proteins, eosinophils produce a range of cytokines, chemokines, growth factors, and lipid mediators.^8^ Through their ability to rapidly release a large range of factors, eosinophils contribute to the immune response against helminths and bacterial infections, not only to physiological responses but also to pathologic inflammation in allergic asthma and other eosinophil-associated diseases.^9,10^

More recently, various tissue-specific functions of eosinophils have been described. For example, intestinal eosinophils support epithelial barrier integrity, antibacterial defence, extracellular matrix (ECM) remodeling, and the T cell compartment.^5,11–14^ In the adipose tissue, eosinophils maintain an anti-inflammatory type 2 immune environment and promote sympathetic nerves needed for healthy metabolic regulation.^15–17^ Eosinophils also play a role in organ morphogenesis in the mammary glands and uterus and contribute to tissue regeneration following injury in the thymus, heart, and liver.^18–22^ These examples underscore that eosinophil biology cannot be reduced to a blunt allergic effector role but must be understood through the lens of organ-specific adaptation.

Tissue adaptation of immune cells occurs over time in response to local niche signals and encompasses transcriptional and metabolic rewiring that leads to stable resident phenotypes and functions. In recent years, a number of studies have observed these processes also in tissue eosinophils. For example, individual niche signals like dietary components or cytokines have been identified that induce eosinophil adaptation. Several studies show that eosinophils isolated from different organs have distinct transcriptional profiles as well as tissue-specific phenotypes and functions.^13,14,23–25^ While eosinophil turnover in the blood and spleen is fast, their lifespan in other tissues is longer.^13,26–28^ This allows time for gradual adaptation, which has been shown to proceed through phenotypically distinct stages with different microanatomical localization.

**Figure 1:**
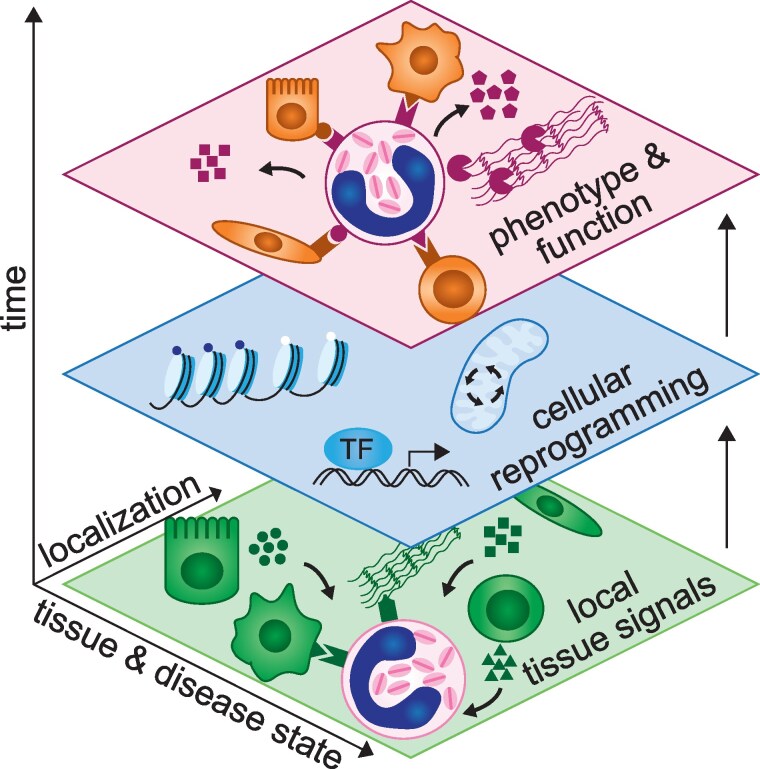
The process of tissue adaptation. Tissue adaptation occurs in response to signals provided by the local microenvironment. These differ not only between tissues but can also vary within microanatomical locations and change with disease state. In response to these local signals, adapting immune cells undergo cellular reprograming, which occurs on the metabolic, epigenetic, and transcriptional level. Adapted immune cells then express a new profile of phenotypic markers and acquire tissue-specific functions.

Understanding tissue-specific eosinophil programs will be essential for designing interventions that selectively modulate pathogenic functions while preserving eosinophil roles in organ homeostasis, immune defence, and repair. In this review, we will examine the mechanisms underlying this adaptation, including the local tissue cues that initiate the process, the transcriptional, epigenetic, and metabolic reprograming that follows, and the resulting phenotypic and functional adaptations that contribute to eosinophil diversity throughout the body (Fig. 1).

### 1.1. Eosinophil tissue adaptation is induced by local microenvironmental factors

Local tissue environments shape immune cell adaptation through a combination of structural, cellular, and molecular cues.^29^ Incoming leukocytes are exposed to new signals in the form of cytokines, chemokines, growth factors, tissue-specific metabolites, and microbial products, which collectively induce organ-tailored transcriptional and epigenetic programs. Epithelial, stromal, endothelial, and neural cells provide signals in the form of survival niches, adhesion landscapes, and spatially restricted signals.

#### 1.1.1. Stromal, neural, and epithelial cells provide local signals

Fibroblasts and other stromal and immune cells are major producers of eotaxins through which eosinophils are recruited into tissues under homeostatic conditions.^30,31^ Moreover, fibroblasts produce ECM components which eosinophils interact with and which may provide further signals for tissue adaptation. For example, adhesion to fibronectin or laminin prolongs eosinophil survival in culture. Increased survival on laminin-coated surfaces is dependent on α6β1 integrin.^32^ Epithelial and stromal cells also produce alarmins like IL-33, which triggers eosinophil adaptation in the colon and promotes a CD80^+^PD-L1^+^ immunoregulatory phenotype.^24^ Eosinophil tissue adaptation is further shaped by signals from the nervous system. Sensory and autonomic neurons express adhesion molecules and chemokine receptors that facilitate direct contact with eosinophils, and several neuropeptides can act as chemoattractants for eosinophils.^12,14,33,34^ Tissue-adapted small intestinal eosinophils express the neuromedin U receptor 1 (NMUR1).^14^ NMUR1 expression makes the cells responsive to the neurotransmitter neuromedin U and is important for maintaining eosinophil numbers in the intestine, promoting their degranulation and for their ability to promote goblet cells during parasitic infection.

Eosinophils also interact with epithelial cells. Coculture with esophageal epithelial cells substantially prolongs eosinophil survival and induces upregulation of CD69.^35^ In the intestine, epithelial cells are the major source of Notch ligands.^36^ Expression of the NOTCH2 receptor on eosinophils plays a key role in eosinophils' adaptation, including tissue-specific phenotypes and functions.^37^ These studies highlight how a range of local signals provided by epithelial cells, neurons, and fibroblasts are key for the adaptation of eosinophils.

#### 1.1.2. Diet and microbiome influence eosinophil tissue adaptation

Nutrient-derived signals were shown to regulate eosinophil adaptation to the small intestine and to influence their spatial localization within the intestinal tissue. Eosinophils enter near the crypts, migrate toward the villi, and differentiate into a distinct villus-resident CD22^hi^ subset. Retinoic acid acts directly on intestinal eosinophils to promote survival during this upward migration, enabling the accumulation of CD22^hi^ eosinophils.^25^ In contrast, high dietary protein or purified amino acids accelerate eosinophil turnover and apoptosis, selectively depleting the villus-resident subset without affecting bone marrow development. These effects were independent of IL-5 or the microbiome, suggesting a direct role for local nutrient-derived signals in shaping both eosinophil function and positioning in the gut. High-fat diet similarly reduces intestinal eosinophil numbers, as well as those in the adipose tissue, though it has not been tested whether this is due to accelerated eosinophil apoptosis.^15,38,39^ The aryl hydrocarbon receptor (AHR) is key for eosinophil adaptation in the intestine.^13^ As a ligand-activated transcription factor, binding of specific ligands such as indole carbinols is required for release from its cytoplasmic chaperone complex and nuclear translocation. AHR ligands in the small intestine are mostly derived from the diet, in particular from cruciferous vegetables.^40^ This provides a mechanism by which dietary components directly drive the adaptation of eosinophils to the intestinal microenvironment.

The intestinal microbiome is also an important regulator of eosinophils. Germ-free (GF) mice of both C57BL/6 and BALB/c backgrounds have approximately two-fold higher frequencies of intestinal eosinophils compared to specific pathogen-free (SPF) controls.^12,41^ Colonization of GF mice with a complex microbiota restores intestinal eosinophil frequencies to levels comparable to those observed in SPF mice, suggesting that eosinophil numbers are acutely regulated by the microbiome. Indeed, GF mice express higher levels of the pro-survival cytokine IL-3 as compared to SPF mice. Intestinal eosinophils in GF mice also have a reduced cytoplasmic granule content,^41^ although it is not clear whether this is specific to the intestine or a systemic effect. GF mice as well as SPF mice treated with broad-spectrum antibiotics show a reduction in the frequency of PD-L1 ^+^ CD80^+^ eosinophils, suggesting that microbial signals induce expression of these tissue adaptation markers.^24^ Recent work further showed that signaling through pattern recognition receptors is required for the induction of AHR expression in eosinophils,^42^ suggesting that the microbiome also promotes the upregulation of this key transcriptional regulator of eosinophil tissue adaptation. Given that AHR limits the lifespan of intestinal eosinophils and their expression of antiapoptotic genes,^13^ it might be partially responsible for the observed lower eosinophil frequencies in SPF compared to GF mice.

#### 1.1.3. Tissue signals change during infection and inflammation

In the context of infection and inflammation, eosinophils respond to the altered tissue environment by changing their phenotype and function. For example, *Nippostrongylus brasiliensis* infection results in increased IL-13 expression in the lung and leads to the induction of CD101 on pulmonary eosinophils.^43^ Likewise, increased IL-18 during allergic asthma promotes the expression of CD274 on eosinophils.^44^ Inflammation also alters eosinophil–neuron interactions. During allergic asthma, airway nerves recruit eosinophils through eotaxins, and adhesion of eosinophils to the nerves promotes eosinophil activation and degranulation.^45,46^ In inflammatory bowel disease, eosinophils increase localization to mucosal nerves, and in Crohn's disease, extend into the muscle layer.^47^ Evidence suggests that tissue-specific programming of immune cells may begin even before they extravasate from the circulation. Endothelial cells interact with immune cells and modulate their activation and functional state.^48,49^ Early coculture studies showed that endothelial cells promote eosinophil survival and activation. Baseline expression of the activation marker CD69 on eosinophils increased from 5% to 30% upon coculture with untreated endothelial cells and further to 50% when endothelial cells were pretreated with IL-1β. These effects were mediated through interactions between intercellular adhesion molecule-1 (ICAM-1) on endothelial cells and CD11b on eosinophils.^48,50^ Therefore, the inflammatory programming of eosinophils may already begin before they enter the tissue. It is clear that inflammation changes the local microenvironment and thereby the signals that eosinophils receive.

#### 1.1.4. Evidence from in vitro studies

Multiple studies have stimulated eosinophils in vitro with different cytokines to analyze the transcriptional, phenotypic, and functional changes.^24,51–54^ These studies clearly show that eosinophils are plastic in their response to different stimuli with distinct transcriptional programs being induced by each cytokine. They further suggest that some of the effects of local tissue-environmental factors on eosinophils may be reproduced and studied in vitro. For example, mouse peritoneal eosinophils treated with either IL-4 or IFNγ induce distinct gene expression signatures, cytokine release, and surface phenotypes.^51^ Eosinophils stimulated with IL-4 upregulate genes related to cell adhesion and migration and reduce oxidative stress response genes. They also upregulate CD101 and CD69 and secrete more CCL24. In contrast, IFNγ induces expression of genes involved in immune defence, upregulates surface CD274, and secretes more CXCL9 and TNFα. Encounter of apoptotic cells, which are engulfed by eosinophils, induces an anti-inflammatory phenotype, which is further enhanced by co-stimulation with IL-4.^52^ At the same time, contact to apoptotic cells downregulates genes induced by IFNγ or stimulation with dead *Escherichia coli* and reduces secretion of inflammatory cytokines TNFα and IL-6. Encounter of apoptotic cells in the context of differing cytokine milieus is likely to happen also in tissue eosinophils, in particular during inflammation. In bone marrow-derived eosinophils, IL-4 and IL-33 induce partially overlapping gene expression signatures.^53^ IL-33 stimulation drives expression of genes involved in cytokine production, immune response, and NFκB signaling and induces production of IL-6 and IL-13. In addition, IL-33 induces an IL-4 feed-forward loop where IL-4 is released from and binds to eosinophils in an autocrine manner to induce STAT-6 phosphorylation and the expression of genes like *Retnla* or *Tfec* and secretion of RELMα and CCL17. IL-33 also induces an eosinophil phenotype characteristic of intestine-adapted eosinophils,^24^ linking in vitro stimulation to tissue eosinophils. Using human blood eosinophils, it was shown that stimulation with IL-4 and IL-5 induces expression of the metabolic enzyme GGT5, needed for the generation of proinflammatory leukotriene D4 and increased leukotriene D4 release.^54^ IL4 also increases expression of the IL-33 receptor (IL1RL1). These reports show how different stimuli can induce distinct gene expression and functional profiles in eosinophils and link these stimuli to phenotypes observed in tissue eosinophils.

**Figure 2:**
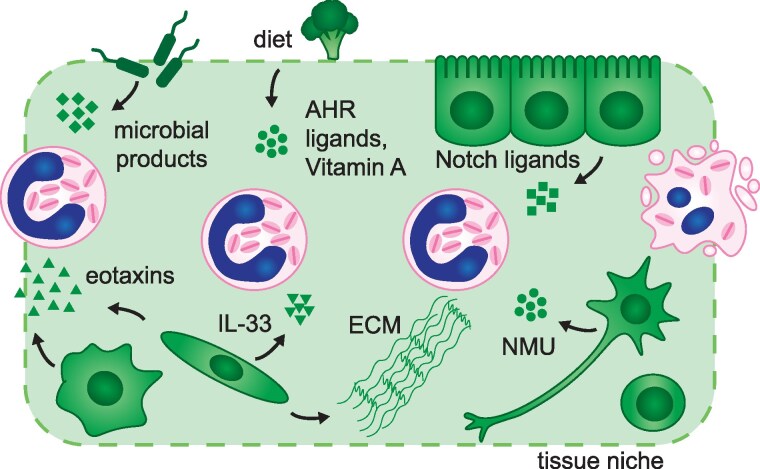
Local tissue signals induce eosinophil adaptation. Following entry into the tissues, eosinophils are exposed to new signals provided by the local microenvironment. While these are not fully understood, some examples in the intestine include microbial products, dietary ligands such as vitamin A and AHR ligands, cytokines, and chemokines produced by fibroblasts or macrophages, neurotransmitters, ECM components, or growth factors. During tissue adaptation, eosinophils may go through several tissue niches, which provide distinct local signals.

Studies to date have discovered a range of local signals that induce tissue-specific phenotypes and functions in eosinophils (Fig. 2). Nevertheless, the picture remains incomplete for intestinal eosinophils and is entirely lacking for most other organs. The relative importance of different local signals, a potential chronological order, how signals interact, and how they are integrated in eosinophils to converge on the induction of the adaptation process are still unknown.

### 1.2. Transcriptional reprograming of eosinophils is coordinated by regulatory networks

During eosinophil development, a dynamic network of transcription factors, including GATA-1, GATA-2, FOG-1, IRF8, C/EBPa, C/EBPe, PU.1, and ID2, regulates eosinophil cell fate and differentiation.^55–57^ Once released from the bone marrow as mature cells, eosinophils continue to change their transcriptional profile. Multiple studies to date have shown that eosinophils from different tissues are transcriptionally distinct from each other and from those found in the blood or bone marrow. Differences between lung and small intestinal eosinophils were first discovered in 2012,^23^ and in recent years, multiple groups have described tissue-specific eosinophil transcriptomes across different organs in mice^13,14,24,58^ and humans.^59,60^ These differences in transcriptomes suggest that eosinophils undergo tissue-specific transcriptional reprograming as part of their adaptation to the local environment—a process that has been described for many types of immune cells, including macrophages, T cells, and innate lymphoid cells and often involves specific transcription factors.^1^ For example, AP-1 family transcription factors are key for the transcriptional program of resident memory T cells in the intestine or skin,^61^ while Runx3 and Nr4a1 regulate homeostatic intestinal macrophages.^62^

For eosinophils, only a few transcription factors in the tissue adaptation process have been identified. Our previous work showed that the eosinophil transcriptome is reprogramed in the small intestine and identified transcriptional regulators that partially control this reprograming.^13^ The AHR is induced in eosinophils after migration into the intestine and regulates part of the intestine-specific transcriptome. Eosinophils lacking AHR showed altered survival, degranulation, immunoregulatory phenotype, as well as ECM adhesion and degradation. A second transcription factor in the same pathway, the AHR repressor (AHRR), regulates a distinct, smaller set of genes in intestinal eosinophils, relating to antimicrobial and innate immune defence.^42^ AHR also regulated the expression of *Clec4a4*, a marker for immunoregulatory intestinal eosinophils^27^ and of *Nmur1*, which was then shown to be important for their promotion of goblet cell differentiation.^14^ Using single-cell RNA sequencing, Gurtner et. al. identified distinct subsets of intestinal eosinophils.^24^ Through prediction of transcriptional regulators, they identified, among others, NF-κB signaling as an upstream regulator of the tissue-adapted CD274 + CD80+ intestinal eosinophil population. Similarly, in human colonic eosinophils, NF-κB and AP-1 complex transcription factors (FOSL1, FOSL2, FOSB) are predicted to regulate specific gene sets.^60^ NOTCH2 was identified as a further transcription factor regulating intestinal eosinophils, promoting the typical intestine-adapted CD11c + CD80+ PD-L1+ CLEC4A4+ phenotype.^37^ While NOTCH2 does not regulate eosinophil cell numbers in the intestine, it increases their migration across the epithelial barrier in a model of oral allergen challenge. Dietary vitamin A can regulate eosinophil survival in the intestine, suggesting a role for retinoic acid receptors (RARs).^25,63^ Small intestinal eosinophils express the ligand-activated transcription factors RARα and RARγ. Inhibition of RARs shortened eosinophil lifespan in the intestine and reduced expression of CD22 as well as the presence of eosinophils within the villi as compared to the crypts. At the same time, retinoic acid increased the viability of small intestinal eosinophils cultured in vitro. While RARα or RARγ were not specifically deleted in eosinophils, this still suggests an important role for these transcription factors in intestinal eosinophils.

**Figure 3:**
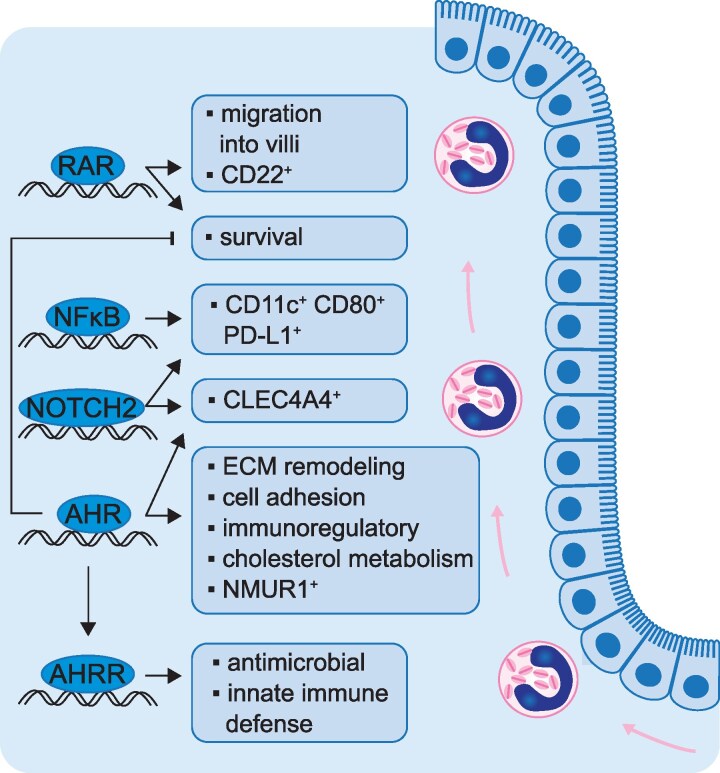
Transcriptional reprograming in the intestine is coordinated by regulatory networks. Several transcription factors involved in eosinophil reprograming in the intestine have been identified, including AHR, AHRR, NOTCH2, NFκB, and RAR. They control partially overlapping phenotypes and functional adaptations, but it is not yet clear how the transcriptional regulators interact with each other.

Not only does the transcriptome of eosinophils differ between tissues, but eosinophils also change their gene expression profiles within tissues over time. This has been demonstrated for the intestine, where eosinophils reside for 2–4 weeks and during this time change their localization as well as transcriptome.^13,24,25,27^ Over their lifetime in the intestine, eosinophils become more immune regulatory, change expression of metabolic pathway genes, downregulate protein translation, and upregulate antimicrobial genes. Taken together, these studies suggest that in the intestine, eosinophil adaptation proceeds over time, involving a network of transcription factors that regulate a broad range of pathways to control the functions of the intestinal tissue-adapted eosinophil (Fig. 3).

In the adipose tissue, eosinophils also acquire a tissue-specific signature that is distinct from those in the blood.^58^ Computational analysis of the data revealed potential upstream regulators, including AP-1 family members FOS, FOSB, JUN, JUNB, JUND, but their potential role in regulating the adipose-specific eosinophil transcriptome was not further studied. The transcription factor KLF3 is expressed in adipose tissue eosinophils, and eosinophils from Klf3-deficient mice show altered gene expression and increased eosinophil numbers in adipose tissue.^64^ However, this was not tested with eosinophil-specific KLF3 deletion, so indirect effects on the observed adipose tissue beiging cannot be excluded. It will be intriguing to see if the transcription factors regulating eosinophils in the adipose tissue are distinct from those in the small intestine.

A recent study has analyzed the transcriptome and open chromatin regions in eosinophils from the esophagus of transgenic mouse models and identified a large set of genes with differentially open chromatin that were also altered transcriptionally in comparison to bone marrow eosinophils.^65^ Eosinophil-specific ATF3 deletion resulted in changed expression of over 400 genes during eosinophilic esophagitis, while there were virtually no differences in control mice. This suggests a critical role for ATF3 in eosinophils during eosinophilic esophagitis only, which does not extend to homeostatic eosinophils. It is not yet clear if the regulation of inflammatory versus homeostatic eosinophils is generally governed by distinct sets of regulatory networks.

The transcription factor networks that drive eosinophil adaptation remain largely unknown for most tissues. However, we are beginning to understand some of the key transcriptional regulators that govern adaptation of intestinal eosinophils (Fig. 3). These known transcription factors only account for some of the observed gene expression differences between intestinal and other tissue eosinophils, suggesting that additional regulators are yet to be discovered. Furthermore, possible interactions of regulatory networks in a temporal or hierarchical manner may exist but have not been studied. For a full mechanistic understanding, it would be key to link local tissue signals to transcriptional regulators and subsequent functional changes in eosinophils.

### 1.3. The tissue environment directly influences eosinophil lifespan

Tissue adaptation is required for cell survival in the local environment, but at the same time, local signals can also affect the lifespan of immune cells. Eosinophil turnover differs dramatically between tissues. Using BrdU labeling, early studies found that eosinophils in the blood and lung were completely replaced with BrdU^+^ cells within 3 days. Within this timeframe, under 10% of thymic and uterine eosinophils became BrdU^+^, and it took 14 days to replace about 80% of small intestinal eosinophils.^26^ This suggested a substantial variation in eosinophil lifespan. A new study tracked eosinophil longevity across different tissues using genetic fate mapping.^28^ They confirmed previous findings that eosinophils in the blood, lung, and spleen are very short-lived. An intermediate lifespan was observed in eosinophils in the colon, thymus, heart, and kidney, and the longest lifespan was found for eosinophils in the small intestine, skin, and adipose tissues. Mechanistically, differences in survival may relate to the expression of antiapoptotic proteins, which are increased in tissue eosinophils.^28^ Interestingly, the lifespan of tissue eosinophils can be manipulated by changing local tissue cues. For example, changing to a high-protein diet decreases eosinophil lifespan in the small intestine^25^ while deletion of AHR, which is activated by dietary ligands, increases eosinophil lifespan in the gut.^13^ The microbiome has a strong effect on eosinophil lifespan as well, as eosinophil turnover is much slower in GF compared to SPF mice.^12^ These studies demonstrate that the microbiome and diet directly affect eosinophil lifespan in the intestine. Although this remains to be tested, it is likely that other tissue signals like growth factors and cytokines will also affect eosinophil longevity within different organs. Importantly, longer lifespans increase the time of residency within the tissue environment. Studies in other immune cells have shown that tissue adaptation occurs gradually over time, which thus allows long-lived cells to take on more specialized adaptations.^3^ For eosinophils, this suggests that populations with long lifespans, such as in the intestine and adipose tissue, may be able to take on unique tissue-specific functions.

### 1.4. Eosinophils take on tissue-specific phenotypes

Over the last decade, many studies have described the varying phenotypes of eosinophils in different tissues or disease models, with a major focus on eosinophils in the intestine and lung.^66^ Recently, high-dimensional characterization of the eosinophil phenotype across a range of tissues yielded new insights into different eosinophil populations.^28^ Cells largely cluster by tissue origin, with eosinophils from some tissues closely resembling each other (such as those from blood, lung, and spleen) while others form distinct tissue-specific clusters (adipose tissue and small intestine). This work also demonstrated that long-lived eosinophils show more distinct phenotypes as compared to short-lived eosinophils, providing clear evidence for a role of the local tissue environment in shaping eosinophil phenotypes over time.^28^

#### 1.4.1. Bone marrow and blood

During eosinophil development in the bone marrow, surface receptors can be used to distinguish eosinophil progenitors and mature eosinophils. Classically, eosinophil progenitors are identified as Lineage^−^ CD34^+^ Sca1^−^ SiglecF^+^ IL-5Rα^+^ CD117^lo^ cells and mature eosinophils as SiglecF^+^ CCR3^+^ CD117^−^ cells.^67^ More recently, detailed analysis revealed phenotypic progression through 4 developmental stages.^57^ All of these are Lineage^−^ CD29^−^ and SiglecF^+^. Progenitors first express high levels of CD117, which is gradually reduced. Mature eosinophils upregulate CCR3 as well as PIR-A/B while losing CD117 expression and are released into the bloodstream. Circulating eosinophils are characterized by expression of CCR3, PIR-A/B, CD11a, CD62L, and integrin α4β7 as well as the absence of tissue adaptation markers.^28^

#### 1.4.2. Lung

It is becoming increasingly clear that at steady state, eosinophils in the lung are located intravascular and only migrate into the parenchyma during infection or inflammation.^28,43^ It is therefore not surprising that lung eosinophils in naïve animals closely resemble circulating eosinophils, with high CD62L expression.^26,68^ In contrast, inflammatory eosinophils located in the lung parenchyma show a different phenotype. They are CD62L^−^, CD101^+^, and SiglecF^hi^.^43,69,70^ A subset of inflammatory eosinophils also expresses low levels of MHC-II, and a fraction of them upregulate CD11c and CD80 while decreasing integrin β7.^68^ Two distinct subpopulations then emerge, defined by their levels of CD11c, integrin β7, and PSGL1 (CD162).^68^ In the context of allergic asthma, lung eosinophils can also upregulate CD274 in response to IL-18 stimulation.^44^ These studies show that the phenotype of eosinophils in the lung is strongly dictated by local inflammation or infection, but not by a tissue adaptation process under homeostatic conditions.

#### 1.4.3. Spleen

Eosinophils in the spleen closely resemble those in the blood, with only small differences in surface receptor expression and transcriptome, and they also lack tissue adaptation markers such as CD11c.^14,24,28,68^ Spleen eosinophils also show the same rapid turnover as those in the blood and lung.^26,28^ Taken together, these observations suggest that eosinophils found in the lung and spleen under homeostatic conditions are recently recruited blood eosinophils that do not undergo any major tissue-specific adaptation.

#### 1.4.4. Intestine

In the small intestine, localization and time of residence are major determinants of the eosinophil phenotype. Newly recruited eosinophils, which first enter through blood vessels in the crypts, phenotypically resemble circulating eosinophils. They express high levels of integrin α4β7 and lack intestine-specific makers.^25,71,72^ As they migrate from the crypts into the villi, they gradually modify their surface receptor expression. First upregulating CD11c, then C-type lectin CLEC4A4, as well as the costimulatory and inhibitory molecules CD80 and PD-L1.^24,26,27,72,73^ Over time, small intestinal eosinophils also upregulate NMUR1 and MHCII.^14,73^ The lectin CD22 is the last to appear on small intestinal eosinophils located at the tip of the villi.^23,25,72^ These findings show that the progressive evolution of the eosinophil phenotype is closely linked to their migration along the villi. Eosinophils have also been found within the epithelial layer of the intestine. This population has increased SiglecF, CD11b, CD11c, and CD80 and decreased MHCII expression compared to lamina propria eosinophils.^73^ Whether intraepithelial eosinophils represent the necessary last stage of the eosinophil lifecycle in the intestine or rather a subpopulation is not clear. Intestinal eosinophils also change their phenotype in response to infection or inflammation, showing increased SiglecF and CD11b expression, signs of degranulation (CD63 expression and loss of SSC), and reduced CLEC4a4 expression.^11,72^ Taken together, these studies identify a clear temporal and spatial pattern of phenotypic adaptation for small intestinal eosinophils.

#### 1.4.5. Adipose tissue

In the adipose tissue, eosinophils are present at high frequencies in mice and humans and have a particularly long lifespan.^15,28,74^ The phenotype of adipose tissue eosinophils has only recently been studied and is characterized by induction of IL33R, CD45RB, MHCII, CD29, and CD90.^28^ In this study, Hu and colleagues demonstrated that adipose tissue eosinophils, alongside those in the small intestine, not only have a particularly long lifespan but also a very distinct phenotype as compared to cells from the blood or lung. It was also shown that sex and the metabolic state of the animal can influence their phenotype. SiglecF expression on adipose tissue eosinophils is higher in males and reduced by high-fat diet.^75^ It is not yet known if additional changes occur in adipose tissue eosinophils in response to high-fat diet or cold exposure. How the adipose tissue eosinophil phenotype changes over the course of their lifespan and if tissue-specific markers are induced subsequently, as was shown in the intestine, has not been studied. Nevertheless, it is already clear that eosinophils undergo phenotypic adaptation in the adipose tissue.

#### 1.4.6. Thymus

Eosinophils also colonize lymphoid organs such as the thymus. Here, the phenotype is dependent on their localization as well as the development stage of the animal. During neonatal development, eosinophils migrate into the thymus. They first appear in the corticomedullary region of the thymus, then gradually move to the medulla. An increasing proportion of these eosinophils express MHCII, which is highest on eosinophils located in the medulla.^76,77^ In adult mice, thymic eosinophils can express MHCII, CD11c, CD80, and lower levels of MHCI, CD30L, and CD86.^20,26,76–78^ Thus, thymus eosinophils adapt to their local microenvironment.

#### 1.4.7. Lymph nodes

In response to local infection or inflammation, eosinophils can migrate to tissue-draining lymph nodes.^79–82^ For example, during infection with *Trichuris muris*, eosinophils accumulate in the medullary region of the mesenteric lymph nodes. These cells express the classic markers of eosinophils (SiglecF, CCR3, CD11b, F4/80), as well as CD11c, Gr-1, and Ly6C.^80^ It has also been shown that eosinophils can induce the expression of CD197 (CCR7), facilitating their migration to the lymph nodes in the context of airway inflammation.^79^

#### 1.4.8. Esophagus

Like in the lung, eosinophils are not present in the esophagus at steady state, but infiltrate during eosinophilic esophagitis. Here, they show distinct transcriptional profiles as compared to blood eosinophils in both mice and humans.^65,83^ They also differ phenotypically from eosinophils in the blood with upregulation of the receptor VSTM1 in humans,^83^ and expression of C3aR1, CD80, ST2, TLR4, and CD44 in the mouse.^65^

**Figure 4:**
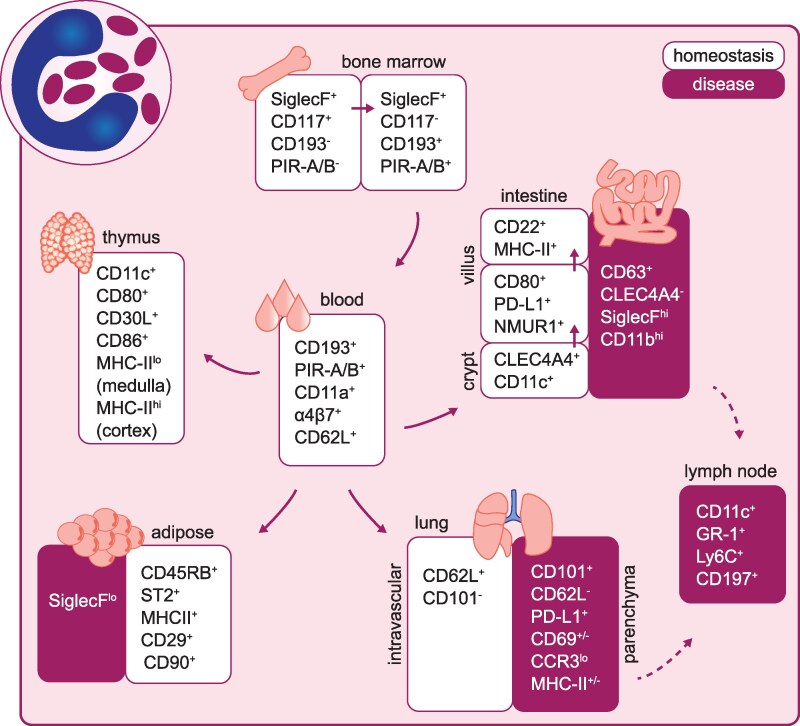
Eosinophils take on tissue-specific phenotypes. Following eosinophil development in the bone marrow, mature eosinophils are released into the bloodstream, expressing characteristic eosinophil markers as well as surface receptors specific to circulating eosinophils. Eosinophils in the lung and spleen closely resemble those in the blood. Inflammation or infection alters the phenotype and localization of lung eosinophils. In the adipose tissue, thymus, and intestine, eosinophils acquire new phenotypic markers. In the intestine, this occurs gradually over time as cells migrate from the crypts into the villi. During infection, eosinophils from the lung and intestine can migrate to draining lymph nodes, again adapting their phenotype.

To date, the phenotype of tissue eosinophils is well characterized in the small intestine and in the inflamed lung, but our knowledge remains limited in most other tissues where eosinophils are present (Fig. 4). For example, phenotypic characterization of eosinophils in the female genital tract, mammary gland, and skin is lacking. From the existing data, it is already clear that eosinophil lifespan and time of residency in the tissue are related to phenotypic changes. Here, clear differences are observed between short-lived blood, lung, and spleen eosinophils, which closely resemble each other and long-lived small intestinal eosinophils that undergo drastic phenotypic changes over time. Longer lifespans result in more time spent in the tissue environment. This is critical for eosinophils to adapt and take on tissue-specific functions. Despite recent studies profiling eosinophil phenotypes across tissues, gaps still remain in our understanding of how these profiles are connected to local tissue signals and the resulting transcriptional reprograming, as only a few direct links in this process have been established.

### 1.5. First insights into metabolic adaptation of tissue eosinophils

In contrast to other cell types, immune cells move from the lymphoid tissues in which they develop to different organs with varying metabolic rates and fuel requirements. To function optimally within the distinct nutrient and signaling landscapes of each tissue, they must undergo metabolic adaptation. Immune cells arriving in non-lymphoid sites such as gut, adipose tissue, or lung rewire their metabolic pathways in response to local availability of glucose, lipids, amino acids, and oxygen, shifting the balance between glycolysis, oxidative phosphorylation, and fatty acid oxidation. This remodeling is tightly linked to transcriptional and epigenetic programs: cues such as hypoxia, growth factors, cytokines, and tissue-derived metabolites engage regulators to couple metabolic state to fate decisions like tissue residency, regulatory versus effector differentiation, and acquisition of trained or exhausted phenotypes. As a result, gene expression of metabolic pathways varies between immune cells.^2,84^ Thus, metabolic fine-tuning is a key feature of tissue adaptation. This process is very well studied in macrophages and T cells as they reside in the majority of tissues and adapt their metabolism to fulfil the specific needs of each tissue.^85^ For example, PPARg regulates lipid metabolism pathways like beta-oxidation of fatty acids in alveolar macrophages, which are necessary for the degradation of surfactants in the alveoli. In contrast, neutrophils, which are short-lived, rely heavily on glycolysis for phagocytosis and neutrophil extracellular trap formation, as it is a faster way of energy production.^86,87^

To date, little is known about eosinophil metabolism, in particular in the context of tissue adaptation. Porter et al. compared neutrophils and eosinophils in human blood and suggested that while both cell types rely on glycolysis, eosinophils exhibit greater metabolic flexibility by also engaging in pathways like glucose oxidation and mitochondrial oxidative phosphorylation.^86^ Upon in vitro activation with IL-5 and GM-CSF, human eosinophils enhance glycolysis as well as mitochondrial respiration.^88^ However, both these studies were focused on blood eosinophils, and only a few studies have examined eosinophil metabolism in tissues. In the intestine, AHR affects the expression of genes in the arachidonic acid metabolism and cholesterol biosynthesis pathways.^13,27^ However, the relevance to their tissue function is not clear. During eosinophil development in vitro, the mitochondria change dynamically in number and morphology.^89^ As eosinophils mature, the number and size of mitochondria decrease while their cristae increase and adopt a lamellar morphology. In vivo, however, eosinophils from tissues show varied mitochondrial morphology with mixed cristae, characterized by both tubular and lamellar cristae within the same mitochondrion.^89^ Although this was not the focus of the study, the structure of mitochondria in eosinophils from different tissues was analyzed. In the lung, eosinophils exhibit mitochondria with 90% lamellar cristae, while those in the large intestine show about 50% lamellar cristae and 50% mixed cristae. This suggests that short-lived lung eosinophils have a mitochondrial morphology similar to mature eosinophils during in vitro differentiation, while eosinophils in the colon with a much longer lifespan show mitochondrial remodeling into mixed cristae.

Immune cell metabolism is not only shaped by the tissue environment but also depends on cell state and inflammatory environment. For example, macrophages adopt a proinflammatory, glycolytic state upon activation and can transition to an anti-inflammatory, mitochondrial phosphorylation-dependent state after the resolution of infection.^90^ In line with this, inflammation also affects the metabolism of eosinophils. In vitro, activation of human eosinophils increases their oxygen consumption rate.^86^ In vivo, injection of IL-33 or allergic asthma models induces an increased glucose uptake by eosinophils in the lung. This increased glucose consumption by activated eosinophils suppresses NK cell function in the lung.^91^ A very similar mechanism was discovered in the skin during infection.^92^ The authors found that during *Leishmania* infection, skin-infiltrating eosinophils upregulate the glucose transporter GLUT-3 and increase their uptake of glucose. This allows them to outcompete Th1 cells for glucose and consequently suppress Th1 effector function. A recent preprint further confirmed that metabolic activity differs between resident and inflammatory eosinophils in an asthma model.^93^ During allergic inflammation, Siglec-F^hi^ lung eosinophils were identified as a glycolytic, metabolically distinct subset. Their activation and survival were regulated by GPR109A and its ligands, including commensal metabolites and vitamin B3.

During *Staphylococcus aureus* infection, a similar shift towards glycolysis occurs, where eosinophils and eosinophil progenitors upregulate glycolytic and reactive oxygen species (ROSs)-related genes. Targeted metabolomics of bone marrow-derived eosinophils generated in vitro further revealed elevated intermediates of glycolysis, the pentose phosphate pathway, and the tricarboxylic acid (TCA) cycle.^94^ Together, these findings show that bacterial skin infection can metabolically reprogram the bone marrow to produce eosinophils with altered metabolic states that support increased energy production, biosynthesis, and proliferation. In another allergic airway inflammation model, iron was shown to promote eosinophil differentiation by upregulating transcription factors critical for lineage commitment and by sustaining mitochondrial metabolic activity. This led to specific shifts in the TCA cycle, with succinate acting as a key metabolite that further supported eosinophil differentiation.^95^ Influenza A virus was shown to directly modulate mouse eosinophil responses. The authors found that virus exposure lowered basal mitochondrial respiration and overall respiratory activity in eosinophils. Maximal respiration and spare respiratory capacity were also reduced in virus-exposed cells.^96^ These data are in line with a reduction of eosinophil mitochondrial mass and cristae number during influenza virus infection.^89^ Cristae remodeling is a consistent feature of eosinophil activation, also observed in models of allergic asthma and parasitic infection, with an increasing percentage of mixed cristae. This might enhance oxidative phosphorylation, support ROS production, and facilitate integration with the TCA cycle and fatty acid oxidation in mature eosinophils.^89,97^ Overall, these studies suggest that in multiple tissues and disease context, inflammatory eosinophils show an altered metabolism with increased glucose uptake, which not only affects the eosinophils themselves but also impacts the metabolism and function of other immune cells in the environment.

Most studies on eosinophil metabolism have focused on blood or in vitro cultured eosinophils, which display metabolic flexibility and utilize both glycolysis and oxidative phosphorylation. Others have focused on inflammation-induced changes in eosinophil metabolism. These studies already demonstrated that eosinophil metabolism is flexible and readily adapted to changing contexts. How homeostatic tissue eosinophils adapt metabolically, however, is unknown. Emerging evidence suggests that mitochondrial remodeling may be involved in this process—the distinct mitochondrial structures observed in tissue-resident eosinophils hint at reliance on oxidative phosphorylation. So far, comparisons of mitochondrial architecture and metabolic pathway use across tissues have not been conducted. To date, no studies have specifically deleted key metabolic genes in eosinophils, which would be needed to provide mechanistic insights into the relevance of different metabolic pathways. Future studies on how eosinophil metabolism is regulated and how it affects eosinophil survival and function in tissues will greatly improve our understanding of these cells.

### 1.6. The relative importance of local niche signals versus trained immunity

Trained immunity is the long-term functional reprograming of innate immune cells by a primary stimulus that, after returning to a nonactivated state, alters their response to a second challenge.^98^ Trained immunity is antigen-independent and mediated through epigenetic, transcriptional, and metabolic reprograming. Although reversible, a defining characteristic is the lasting nature of these changes, which can be maintained for several months or possibly even years. Epigenetic reprograming can occur not only in peripheral immune cells such as tissue macrophages but also in bone marrow progenitors. As a result, trained immunity can last much longer than the lifespan of individual immune cells.^99^ Trained immunity can also occur in the context of type 2 immune responses, such as allergic asthma. In both patients and animal models, monocyte-derived macrophages display epigenetic reprograming, increased chemokine and eicosanoid production, and altered metabolism.^100^

In multiple models, type 2 immune challenges increase eosinophilopoiesis and eosinophil numbers in peripheral tissues, which causes a heightened eosinophil response and worsened pathology in response to secondary challenges in different tissues. Allergen exposure at one site, for example, the skin, elicits systemic eosinophilia and predisposes for increased eosinophil responses at distinct sites, such as a subsequent allergen challenge in the intestine.^101^ However, whether eosinophils are also phenotypically or functionally different was not investigated. Infection with *N. brasiliensis*, which occurs in the lung and intestine, induces lasting eosinophilia at the sites of infection but also in peripheral tissues, including in the female genital tract.^70,102^ Prior *N. brasiliensis* infection worsens pathology in a subsequent vaginal herpes simplex virus 2 infection. This pathology is reduced by blocking IL-5 or depleting eosinophils, suggesting that eosinophils are a key driver of disease.^102^ However, it is not clear whether the increased pathology was simply a consequence of the increased number of eosinophils or if indeed their function was altered due to the primary helminth infection. Nematode-infected mice also acquire resistance to subsequent infection with an unrelated nematode 1 month later, in an eosinophil-dependent manner.^103^ While these studies show that eosinophils are implicated in altered responses to secondary challenges, none of them analyze potential changes to bone marrow progenitors or lasting changes to eosinophils function.

A recent study delineated the development of eosinophils in the BM through 4 stages from early progenitors to mature eosinophils.^57^ As in previous studies, parasitic infection, allergic asthma models, or injection of IL-33 lead to eosinophil progenitor expansion and peripheral eosinophilia. Jorssen and colleagues found that eosinophils continue to mature along the four stages after induction of eosinophilia, but proliferation of progenitor stages is increased and continues into stage 3 and to some extent into mature eosinophils of stage 4. As proliferation was prolonged, expression of differentiation and maturation genes was delayed during eosinophilia. Transcriptional differences were retained in mature eosinophils, with increased expression of *Junb* and *Irf8* in steady state eosinophils and increased expression of *Epx* and *Gata2* in IL-33-treated mice. This suggests that type 2 inflammation can alter not only the number of eosinophils that are released from the bone marrow but also changes their properties and provides an example of transcriptional changes occurring during trained immunity. As a result, these eosinophils generated in an inflammatory environment may respond differently to local tissue signals or secondary challenges, although this was not directly tested. Very recently, it was shown that infection can induce lasting epigenetic reprograming of eosinophils.^94^ Cutaneous infection with *Staphylococcus aureus* leads to increased inflammation in a subsequent allergic response to house dust mite. The bacterial infection causes reprograming of eosinophils in the bone marrow, altering their epigenetic landscape. This reprograming persisted in bone marrow-derived eosinophils ex vivo, as well as following adoptive bone marrow transfer into irradiated recipient mice. This suggests that eosinophils can indeed acquire trained immunity, leading to lasting changes in eosinophil progenitors or hematopoietic stem and progenitor cells, which in turn alter the way mature eosinophils respond to secondary challenges. It seems likely that trained immunity can also affect the tissue adaptation process of eosinophils; however, this remains to be studied. Currently, it is unknown whether local tissue signals can override or interact with epigenetic changes induced by trained immunity.

## 2. Concluding remarks

Eosinophil tissue adaptation has only recently emerged as a distinct focus of research, and many aspects of this process remain unresolved. During the last 5 years, studies have identified local cues and transcriptional regulators that contribute to eosinophil adaptation, mapped phenotypic changes across space and time and linked eosinophil function to tissue and disease contexts. Although the overall picture remains incomplete, a blueprint for the tissue adaptation process is emerging. Upon tissue entry, eosinophils encounter a range of local signals. These induce transcriptional regulators and possibly epigenetic remodeling and metabolic changes, which in turn change eosinophil phenotype and functions. Key open questions remain on the relative contribution and hierarchy of local signals and transcriptional regulators as well as the importance of systemic changes in eosinophil progenitors versus local adaptation. To date, most findings are based on mouse models. Future work should aim to map this process in humans and determine its functional relevance in health and disease.
